# Genetic Structure of *Apis cerana* Populations from South Korea, Vietnam and the Russian Far East Based on Microsatellite and Mitochondrial DNA Polymorphism

**DOI:** 10.3390/insects13121174

**Published:** 2022-12-17

**Authors:** Milyausha Kaskinova, Luisa Gaifullina, Rustem Ilyasov, Arkady Lelej, Hyung Wook Kwon, Pham Hong Thai, Elena Saltykova

**Affiliations:** 1 Institute of Biochemistry and Genetics, Ufa Federal Research Center, Russian Academy of Sciences, Prospekt Oktyabrya 71, 450054 Ufa, Russia; 2 Scientific and Educational Center, Bashkir State Agrarian University, 50-Letiya Oktyabrya Str. 34, 450001 Ufa, Russia; 3 Department of Genetics and Biotechnology, Vavilov Institute of General Genetics, Russian Academy of Sciences, Gubkina Str. 3, 119333 Moscow, Russia; 4 Federal Scientific Center of the East Asia Terrestrial Biodiversity, Prospekt 100-let Vladivostoka, 159, 690022 Vladivostok, Russia; 5 Department of Life Sciences and Convergence Research Center for Insect Vectors, Incheon National University, 119 Academy-ro, Yeonsu-gu, Incheon 22012, Republic of Korea; 6 Research Center for Tropical Bees and Beekeeping, Vietnam National University of Agriculture, Trau Quy, Gia Lam, Hanoi 10000, Vietnam

**Keywords:** *Apis cerana*, microsatellite loci, *tRNAleu-COII* locus, genetic structure, haplotype diversity

## Abstract

**Simple Summary:**

The oriental honey bee, *Apis cerana*, is an important pollinator of crops and natural phytocoenosis in Asia. Natural barriers in the form of forests, mountains, rivers and seas have created the prerequisites for the formation of subspecies and ecotypes of the *A. cerana*. We analyzed the genetic structure of *A. cerana* samples from the Russian Far East, Vietnam and South Korea. High haplotype diversity was found in all samples of *A. cerana* based on analysis of the *tRNAIeu-COII* locus sequences. The found polymorphisms can be used as genetic markers to differentiate subspecies of *A. cerana*.

**Abstract:**

In this article, we present the results of the genetic analysis of *Apis cerana* samples from the Russian Far East, South Korea and Vietnam. An analysis of the polymorphism of seven microsatellite loci and an assessment of the haplotype diversity of the mtDNA *tRNAleu-COII* locus were performed. A fragment of about 431 bp in *tRNAleu-COII* was sequenced. The analysis showed the presence of 14 haplotypes, while the predominant haplotype was Japan1. Microsatellite data revealed two differentiated clusters. The first cluster contained tropical climate *A. cerana* samples from Vietnam, and the second cluster combined temperate climate *A. cerana* samples from the Russian Far East and South Korea.

## 1. Introduction

The eastern wax bee (*Apis cerana* F.) plays an important role in local ecosystems and agriculture in Asia. The natural range of *A. cerana* has embraced almost all Asian countries, from Indonesia to 47°54′ N latitude in Khabarovsky Krai of Russia [1,2,3]. In the Russian Far East, there is only a feral population of *A. cerana* with an undefined number of colonies [4]. Populations of *A. cerana* are separated from each other by natural barriers in the form of mountains and forests, rivers and seas, which creates prerequisites for the formation of local ecotypes and subspecies. Ecotypes and subspecies of honey bees can be differentiated using morphometric and genetic methods. Morphometric and molecular genetic methods have been developed and effectively used to differentiate subspecies of the European honey bee *A. mellifera* [5]. These methods are also used in *A. cerana* research. 

Based on morphometric differences, Yang (1984) [6] identified four geographic races of *A. cerana*: Eastern, Southern Yunnan, Aba and Xizang (or Tibet). Later, his data were confirmed by Peng et al. (1989) [2]. Radloff et al. (2005) [3] performed morphometric analyses on 557 colonies of *A. cerana* from all of southern mainland Asia extending from Afghanistan to Vietnam south of the Himalayas. To analyze genetic differences between *A. mellifera* subspecies, the polymorphism of the mitochondrial marker, the locus between genes *transfer RNA Leu* and *cytochrome oxidase subunit 2* (*tRNAleu-COII*) [7], and the polymorphism of microsatellite loci of nuclear DNA [8] are usually used. The same markers were also used to analyze *A. cerana* populations. For example, Tan et al. (2007) [9] identified nine *tRNAleu-COII* haplotypes in *A. cerana* populations from China. Zhao et al. (2014) [10], based on the analysis of the mtDNA *COX1* locus, identified 57 haplotypes in 360 colonies of *A. cerana* from 12 geographical populations in China. Lee et al. (2016) [11] estimated the genetic diversity of *A. cerana* population in Korea based on *tRNAleu-COII* locus polymorphism. Xu et al. (2013) [12] with both mitochondrial *tRNAleu-COII* sequences and five microsatellite loci showed that *A. c. cerana* population from Damen Island significantly differentiated from its adjacent mainland populations. Yu et al. (2019) [13] showed the genetic differentiation of *A. cerana* inhabiting the long and narrow alpine valleys of the Qinghai–Tibet Plateau using 31 microsatellite loci and mitochondrial *tRNAleu-COII* fragments. In addition, genome-wide studies of *A. cerana* have recently been performed—Fang et al. (2022) [14] showed that Chinese *A. c. cerana* populations in plains exhibited higher genetic diversity than in mountain areas.

In the present study, a set of seven microsatellite loci and mitochondrial *tRNAleu-COII* locus were used to analyze a genetic structure of *A. cerana* populations from the Russian Far East, South Korea and Vietnam. The aim of this work is to establish whether there are genetic differences between geographically distant of *A. cerana* populations from the Far East, South Korea and Vietnam. 

## 2. Materials and Methods

### 2.1. Sampling and DNA Extraction

Samples of *A. cerana* were collected from the Russian Far East (FE, N = 16 colonies), South Korea (SK, N = 16) and Vietnam(Vn, N = 14). All samples from Vietnam were collected in Ha Noi city: eleven samples were previously identified using morphometric methods [15] as *A. c. indica* and three as *A. c. cerana* (these three colonies were brought from Dong Van district, Ha Giang province). Samples from South Korea were collected from six countries/cities: Incheon (N = 7), Gangneung (N = 1), Hongcheon (N = 1), Chungju-si (N = 5), Sangju (N = 1) and Okcheon (N = 1). Samples from the Far East were collected in Primorsky Krai: in Khasansky district (N = 10), Terneysky district (N = 1) and Vladivostok (N = 5). Workers were collected from managed (South Korea, Vietnam) or wild (Far East) colonies into 96% ethanol. Total DNA was extracted from the thoraces of worker bees from each colony using the GeneJet kit (Thermo Scientific, Waltham, MA, USA).

### 2.2. tRNAleu-COII Locus Analysis

The *tRNAleu-COII* intergenic locus of the mitochondrial genome was amplified using primers (5′-TCTATACCACGACGTTATTC-3′) and (5′-GATCAATATCATTGATGACC-3′). PCR were performed in a final volume of 20 μL: 15 μL sterile ddH_2_O, 2 μL of 10 × PCR Buffer, 0.4 μL dNTP, 0.6 μL each primer (10 pmol/μL), 0.3 μL Taq DNA polymerase and 2 μL DNA template. PCR conditions: initial denaturation at 94 °C for 5 min, followed by 30 cycles of denaturation at 94 °C for 30 s, annealing at 50 °C for 30 s and elongation at 72 °C for 1 min with a final elongation at 72 °C for 10 min. PCR products were examined on 8% polyacrylamide gels (PAAG) stained with ethidium bromide. The gels were visualized in a Gel Doc™ XR+ photosystem (BioRad, Hercules, CA, USA).

Sequencing of the amplified fragments was carried out using the Applied Biosystem sequencer at the Syntol Company (Moscow, Russia). Each sample was sequenced twice. Nucleotide sequences were manually edited in Mega 5.2 software [16] to produce the consensus sequences, which were then aligned with previously published sequences using the Clustal W algorithm. The phylogenetic tree was constructed using the Maximum Likelihood method based on the Kimura 2-parameter model in Mega 5.2. *tRNAleu-COII* locus sequences are available at the link https://doi.org/10.6084/m9.figshare.21393201.v1 (accessed on 18 July 2022). We also downloaded 287 previously published *A. cerana* *tRNAleu-COII* sequences (8 PopSets) from the National Center for Biotechnology Information (“http://www.ncbi.nlm.nih.gov/ (accessed on 18 July 2022)”). The haplotype network was predicted using a median-joining algorithm using the PopArt v1.7 software package (“https://popart.maths.otago.ac.nz/ (accessed on 20 July 2022)”).

### 2.3. Analysis of Microsatellite Loci

A set of microsatellite markers originally developed for *A. mellifera* [8] were prescreened and seven (microsatellite loci Ap243, 4a110, A24, A113, A88, Ap049, and Ab124) showed polymorphism in *A. cerana* samples and were used in this study. Some microsatellite markers (*A43*, *Ap226*, *Ap256*, *A079*) were not polymorphic, some (*A8*, *Ap307*, *A008*, *A007*, *Ap001*, *Ap068*, *Ap207*, *Ap289*) did not form a PCR product in *A. cerana*. 

PCR was performed in a volume of 20 μL: 15 μL sterile ddH_2_O, 2 μL of 10 × PCR Buffer, 0.4 μL dNTP, 0.6 μL each primer (10 pmol/μL), 0.3 μL Taq DNA polymerase, and 2 μL DNA template. PCR conditions: initial denaturation at 94 °C for 5 min, then 25 cycles of denaturation at 94 °C for 30 s, annealing at 55 °C for 30 s, and elongation at 72 °C for 1 min with a final elongation at 72 °C for 10 min. Amplification products were visualized using electrophoresis in 8% PAAGs followed by detection in the Gel Doc XR+ photosystem (BioRad, Hercules, CA, USA).

To determine the genetic structure of *A. cerana* samples, the Structure 2.3.4 program was used with a given number of clusters from 1 to 10. The number of intended groups (K) was calculated in Structure Harvester. The analysis was performed using the Admixture model with information on the geographical location of the samples (LocPrior) and with Burnin Period and MCMC equal to 10,000 and 100,000 repetitions, respectively. The results of analysis were processed in CLUMPP 1.1.2 using the FullSearch algorithm. 

## 3. Results

### 3.1. Haplotype Diversity Based on Polymorphism of tRNAleu-COII Locus

**Figure 1:**
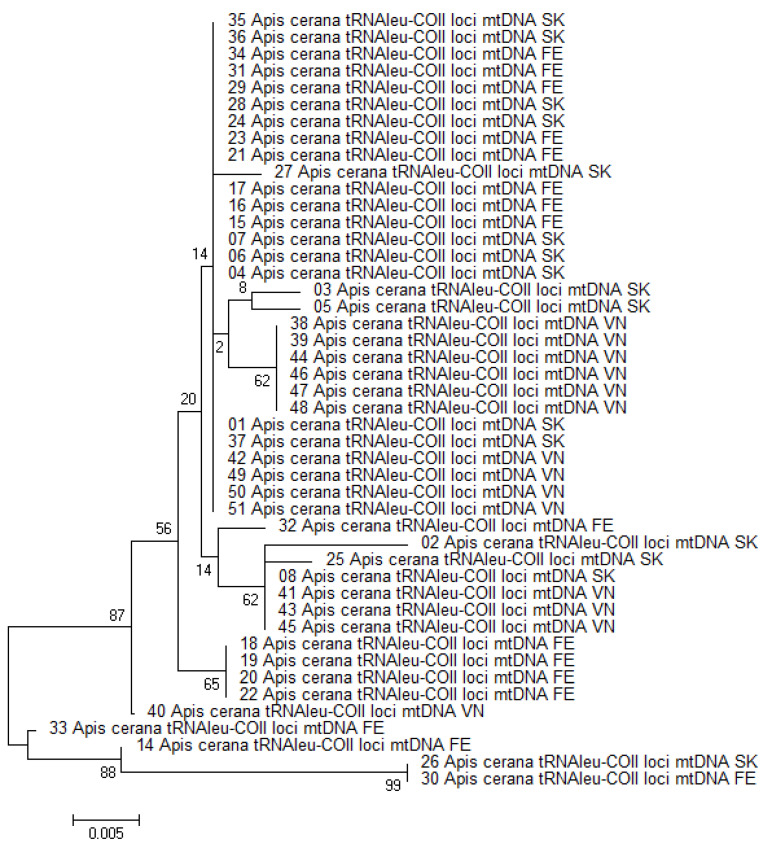
Phylogenetic tree of mtDNA *tRNAleu-COII* locus of 46 *A. cerana* samples. Vn—Vietnam samples, SK—South Korea samples, FE—Far East samples.

The obtained 46 *tRNAleu-COII* locus sequences have a length of 431 bp and 14 haplotypes (Figure 1), of which 10 haplotypes were found in the South Korean sample of *A. cerana*, 6 haplotypes were found in the Russian Far East sample and 4 were found in the Vietnamese sample. Six haplotypes from SK, three from FE and one from Vn were unique (we encountered them once). 

Table 1 presents the characteristics of 287 previously published *A. cerana* *tRNAleu-COII* sequences from NCBI. The maximum length of the fragment of this intergenic locus was recorded in PopSet 1593526727 (757 bp), and the smallest length was recorded in 299829809 (357 bp). The longer the sequence, the more haplotypes were found in the sample. The number of detected haplotypes depended not only on the size of the *tRNAleu-COII* fragment. For example, Takahashi et al. (2007) [17], based on an analysis of 470 colonies of *A. cerana* from Japan, identified only three haplotypes (*tRNAleu-COII* fragment size 774 bp), whereas Gong et al. (2018) [18], having analyzed 1518 colonies from 11 provinces of China, revealed 111 haplotypes (*tRNAleu-COII* fragment size 760 bp).

To compare these sequences, we aligned them and left only the part that matched for all sequences. It was a 241–243 bp fragment. A total of 69 haplotypes were released. Seven new *tRNAleu-COII* haplotypes were detected in our study (four from SK, and three from FE). The majority (21 sequences) of our 46 *A. cerana* sequences of the *tRNAleu-COII locus* belonged to the Japan1 haplotype (9 samples from SK, 8 samples from FE, and 4 samples from VN) and the Korea14 haplotype (four samples from FE). Previously, Tan et al. (2007) [9] reported that most *A. cerana* samples on Hainan island belonged to the haplotype Japan1. These 69 haplotypes differed from the Japan1 haplotype by 1 to 12 substitutions. The greatest differences (12 substitutions) were found with haplotype H3 (DQ381965.1). The H3 haplotype also included two samples from our study, which formed a separate branch on the phylogenetic tree (samples 26 *Apis cerana* *COI-COII* mtDNA SK and 30 *Apis cerana* *COI-COII* mtDNA FE in Figure 1). One sample from SK belonged to haplotype CMHap3 (MH670658.1), one sample from Vn belonged to haplotype CMHap4 (MH670659.1) and six samples from Vn belonged to haplotype CMHap32 (MH670687.1). One sample from SK and three samples from Vn belonged to haplotype CMHap36 (MH670691.1).

**Figure 2:**
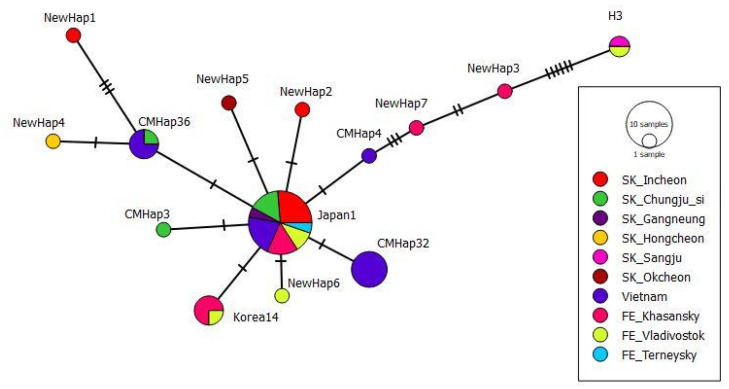
Median-joining networks among the tRNAleu-COII haplotypes of Apis cerana.

Based on the analysis of *tRNAleu-COII* haplotype diversity, we found that, despite the large distance, for all three samples, haplotype Japan1 was predominant (Figure 2). At the same time, a high frequency of the Korea14 haplotype became a distinctive feature for the sample from the Far East, and for the sample from Vietnam, the CMHap32 haplotype was found in of *A. c. indica* colonies.

### 3.2. Genetic Structure of Apis cerana Samples from the Russian Far East, South Korea and Vietnam

**Figure 3:**
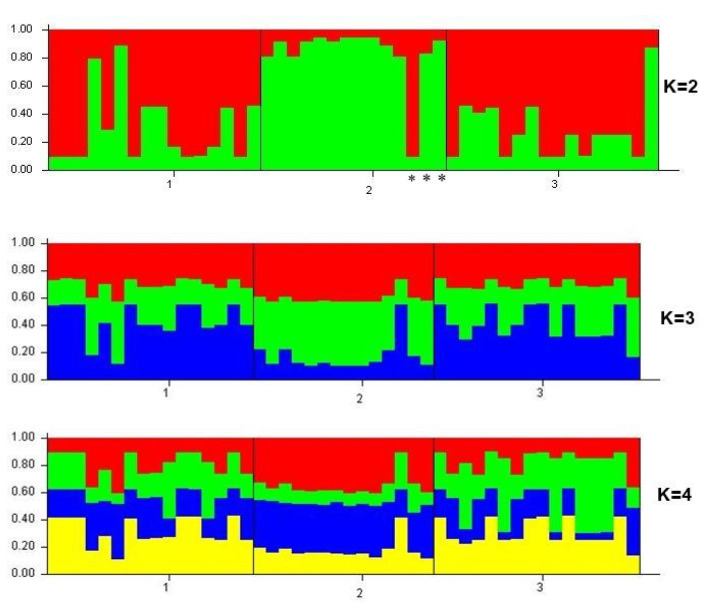
The genetic structure of the studied *Apis cerana* samples: 1—sample from South Korea, 2—samples from Vietnam (subspecies *A. c. indica*, except for specimens marked *—*A. c. cerana*) and 3—samples from the Russian Far East. K is the inferred number of clusters.

The Bayesian approach implemented by the Structure 2.3.4 software was used to cluster individuals into putative populations (K) and determine the genetic structure of the study samples. Analysis of the structure output data in Structure Harvester showed that the total sample consists of two main clusters (K = 2, delta K = 13.948160). The first cluster was represented by SK and FE, and the second cluster included sample Vn (excluding one colony). Figure 3 shows a plot-graph reflecting the structure of populations at K = 2, 3 and 4. A clearer differentiation into clusters was observed at K = 2.

Despite the small sample size, there was a division into two clusters. We observed differentiation of the *A. c. indica* selected in Vietnam from *A. cerana* colonies selected in South Korea and the Far East. Thus, microsatellite data analysis showed that samples from Vietnam may indeed belong to the *A. c. indica*.

## 4. Discussion

The aim of this work was to establish whether there are genetic differences between geographically distant of *A. cerana* populations from the Far East, South Korea and Vietnam. In total, 14 *tRNAleu-COII* haplotypes were identified during the analysis of 46 sequences with a fragment size of 431 bp. As in previous studies [9,11,17], Japan1 was shown to be the predominant haplotype. In the sample from the Far East, the Korea14 haplotype was found with high frequency; in the samples from SK and Vn, it was completely absent. While in the Vietnamese sample CMHap32 was the predominant haplotype. When evaluating haplotypes for the *tRNAleu-COII* locus, the following problem was identified: the authors of the studies used different fragments of this locus, which is due to the sample preparation and capabilities of the sequencer used. Accordingly, the longer the fragment, the more likely it is to identify new haplotypes.

Analysis of the polymorphism of microsatellite loci showed that the studied samples form two clusters. One of the clusters was formed by samples from South Korea and the Far East, and the other was formed by a sample from Vietnam. Most of the samples from Vietnam (11 out of 14) were previously identified as *A. c. indica* based on morphometric methods [15]. Our data (both analysis of microsatellite loci and analysis of *tRNAleu-COII* haplotype diversity) confirmed that it may belong to this subspecies. Thus, this set of microsatellite loci can potentially be used to differentiate *A. c. indica*. However, this requires further research with an increase in the number of samples and expansion of the geography of sampling. The genetic structure of samples from the Far East and South Korea was uniform. Using a selected set of microsatellite loci, we were unable to identify differences between them. 

According to Ruttner (1988), *A. cerana* phenotypically divided into four subspecies/morphoclusters: *A. c. cerana* (in Afghanistan, Pakistan, India, China, Taiwan, Korea and the Russian Far East), *A. c. indica* (in southeast Asia and southern India) and *A. c. himalaya* (from the Himalaya region to Yunnan in China) and *A. c. japonica* (Japan). Radloff et al. (2010) [19] performed morphometric analyses of *A. cerana* populations across its full geographical range and showed that they are subdivided into six morphoclusters. Similar to studies of *A. mellifera* subspecies [20], studies looking for genetic differences between *A. cerana* subspecies are few and are mainly based on the analysis of polymorphisms of mtDNA genes [11,21]. According to Radloff et al. (2010), *A. cerana* populations from Vietnam belong to morphocluster IV (previously named *A. c. indica* and *A. c. javana*). Whereas populations from South Korea and the Far East belong to morphocluster I (previously named as *A. skorikovi*, *A. c. abansis*, *A. c. cerana*, *A. c. indica*, *A. c. japonica*, *A. c. javana*, etc.). Thus, we see that there is still no single approach to the classification of *A. cerana* subspecies and further studies at the genetic level are required. The accurate identification of the *A. cerana* subspecies will allow for the preservation of the unique gene pool of the local subspecies.

## Figures and Tables

**Table 1:** *Apis cerana tRNAleu-COII* sequences from the NCBI and this study.

NCBI PopSet id	b.p.	N	Number of Haplotypes
2178350964	414	2	2
1593526727	757	72	72
1368163505	414	10	10
1368162158	414	2	2
727929638	470	184	10
87477350	432	3	3
299829809	357	10	10
298108794	414	4	4
This study	431	46	14

## Data Availability

*tRNAleu-COII* locus sequences are available at the link https://doi.org/10.6084/m9.figshare.21393201.

## References

[1] Ruttner F. (1988). Apis cerana Fabricius 1793:327. Biogeography and Taxonomy of Honeybees.

[2] Peng Y.S., Nasr M.E., Locke S.J. (1989). Geographical races of *Apis cerana* Fabricius in China and their distribution. Review of recent Chinese publications and a preliminary statistical analysis. Apidologie.

[3] Radloff S.E., Hepburn R.H., Hepburn C., Fuchs S., Otis G.W., Sein M.M., Aung H.L., Pham H.T., Tam D.Q., Nuru A.M. (2005). Multivariate morphometric analysis of *Apis cerana* of southern mainland Asia. Apidologie.

[4] Pesenko Y.A., Lelej A.S., Radchenko V.G., Filatkin G.N. (1989). Chinese wax bee *Apis cerana cerana* F. (Hymenoptera, Apidae) the Far East of the USSR. Entomol. Rev..

[5] Evans J.D., Schwarz R.S., Chen Y.P., Budge G., Cornman R.S., De la Rua P., de Miranda J.R., Foret S., Foster L., Gauthier L. (2013). Standard methods for molecular research in *Apis mellifera*. J. Apic. Res..

[6] Yang G.H. (1984). The survey of the resource of the Chinese honey bee. Zhongguo Yangfeng.

[7] Garnery L., Solignac M., Celebrano G., Cornuet J.-M. (1993). A simple test using restricted PCR-amplified mitochondrial DNA to study the genetic structure of *Apis mellifera* L. Experientia.

[8] Solignac M., Vautrin D., Loiseau A., Mougel F., Baudry E., Estoup A., Garnery L., Haberl M., Cornuet J.-M. (2003). Five hundred and fifty microsatellite markers for the study of the honeybee (*Apis mellifera* L.) genome. Mol. Ecol. Notes.

[9] Tan K., Warrit N., Smith D.R. (2007). Mitochondrial DNA diversity of Chinese *Apis cerana*. Apidologie.

[10] Zhao W., Tan K., Zhou D., Wang M., Cheng C., Yu Z., Miao Y., He S. (2014). Phylogeographic analysis of *Apis cerana* populations on Hainan Island and southern mainland China, based on mitochondrial DNA sequences. Apidologie.

[11] Lee J.Y., Wang A.R., Choi Y.S., Thapa R., Kwon H.W., Kim I. (2016). Mitochondrial DNA variations in Korean *Apis cerana* (Hymenoptera: Apidae) and development of another potential marker. Apidologie.

[12] Xu X., Zhu X., Zhou S., Wu X., Zhou B. (2013). Genetic differentiation between *Apis cerana cerana* populations from Damen Island and adjacent mainland in China. Acta Ecol. Sin..

[13] Yu Y., Zhou S., Zhu X., Xu X., Wang W., Zha L., Wang P., Wang J., Lai K., Wang S. (2019). Genetic Differentiation of Eastern Honey Bee (*Apis cerana*) Populations across Qinghai-Tibet Plateau-Valley Landforms. Front. Genet..

[14] Fang F., Chen X., Lv J., Shi X., Feng X., Wang Z., Li X. (2022). Population Structure and Genetic Diversity of Chinese Honeybee (*Apis cerana cerana*) in Central China. Genes.

[15] Thai P.H., Nguyen T.H., Toan T.V., Jung C. (2018). Apis cerana Beekeeping and Sacbrood Disease Management in Vietnam: Review. J. Apic..

[16] Tamura K., Stecher G., Kumar S. (2021). MEGA11: Molecular Evolutionary Genetics Analysis Version 11. Mol. Biol. Evol..

[17] Takahashi J., Yoshida T., Takagi T., Akimoto S., Wood K.S., Deowanish S., Hepburn R., Nakamura S., Matsuka M. (2007). Geographic variation in the Japanese islands of *Apis cerana japonica* and in *A. cerana* populations bordering its geographic range. Apidologie.

[18] Gong X., Zhao W., Zhou D., Zhang X., Wang M., Dong K., He S. (2018). Genetic variation and population structure of *Apis cerana* in northern, central and southern mainland China, based on *COXI* gene sequences. J. Apic. Res..

[19] Radloff S.E., Hepburn C., Hepburn H.R., Fuchs S., Hadisoesilo S., Tan K., Engel M.S., Kuznetsov V. (2010). Population structure and classification of *Apis cerana*. Apidologie.

[20] Momeni J., Parejo M., Nielsen R.O., Langa J., Montes I., Papoutsis L., Farajzadeh L., Bendixen C., Cauia E., Charriere J.-D. (2021). Authoritative subspecies diagnosis tool for European honey bees based on ancestry informative SNPs. BMC Genom..

[21] lyasov R.A., Youn H.G., Lee M., Kim K.W., Proshchalykin M., Lelej A.S., Takahashi J., Kwon H.W. (2019). Phylogenetic relationships of russian Far-east *Apis cerana* with other north asian populations. J. Apic. Sci..

